# DEMETER DNA demethylase reshapes the global DNA methylation landscape and controls cell identity transition during plant regeneration

**DOI:** 10.1186/s12864-024-11144-x

**Published:** 2024-12-23

**Authors:** Seunga Lee, Soon Hyung Bae, Yunji Jeon, Pil Joon Seo, Yeonhee Choi

**Affiliations:** 1 Department of Biological Sciences, Seoul National University, Seoul, Korea; 2 Research Center for Plant Plasticity, Seoul National University, Seoul, Korea; 3 Department of Chemistry, Seoul National University, Seoul, Korea; 4 Plant Genomics and Breeding Institute, Seoul National University, Seoul, Korea

**Keywords:** *Arabidopsis*, DNA methylation, DEMETER (DME) DNA demethylase, Somatic cell reprogramming, In vitro plant regeneration, RNA-directed DNA methylation (RdDM)

## Abstract

**Background:**

Plants possess a high potential for somatic cell reprogramming, enabling the transition from differentiated tissue to pluripotent callus, followed by the formation of de novo shoots during plant regeneration. Despite extensive studies on the molecular network and key genetic factors involved in this process, the underlying epigenetic landscape remains incompletely understood.

**Results:**

Here, we explored the dynamics of the methylome and transcriptome during the two-step plant regeneration process. During the leaf-to-callus transition in *Arabidopsis* L*er*, CG methylation shifted across genic regions, exhibiting a similar number of differentially methylated regions (DMRs) for both hypo- and hypermethylation. Pericentromeric regions underwent substantial CG and extensive CHH hypomethylation, alongside some CHG hypermethylation. Upon shoot regeneration from callus, genic regions displayed extensive reconfiguration of CG methylation, while pericentromeric methylation levels highly increased across all cytosine contexts, coinciding with the activation of the RNA-directed DNA methylation (RdDM) pathway. However, mutation in *DEMETER* (*DME*) DNA demethylase gene resulted in significant genic CG redistribution and global non-CG hypomethylation in pericentromeric regions, particularly during shoot regeneration. This non-CG hypomethylation observed in *dme-2* mutants was, at least partly, due to RdDM downregulation. The *dme-2* mutants affected gene expression involved in pluripotency and shoot meristem development, resulting in enhanced shoot regeneration through a reprogrammed state established by pericentromeric hypomethylation compared to wild type.

**Conclusion:**

Our study demonstrates epigenetic changes, accompanied by transcriptome alterations, during pluripotency acquisition (leaf-to-callus) and regeneration (callus-to-de novo shoot). Additionally, it highlights the functions of the DME demethylase, particularly its close association with the RdDM pathway, which underlies pericentromeric non-CG methylation maintenance. These results provide important insights into the epigenetic reconfiguration associated with cell identity transition during somatic cell reprogramming.

**Supplementary Information:**

The online version contains supplementary material available at 10.1186/s12864-024-11144-x.

## Background

Plant somatic cells exhibit a remarkable capacity to undergo cellular reprogramming and regenerate into new organs. This ability has been exploited to in vitro tissue culture, in which a pluripotent cell mass called callus is induced from tissue explants on auxin-rich callus-inducing medium (CIM), which then allows de novo organogenesis on shoot-inducing medium (SIM) [1]. Callus formation begins at pericycle-like cells, with the proliferation of the root founder cells to form a cell mass that resembles the lateral root primordia on CIM [2, 3]. The key factors establishing root founder cells, WUSCHEL-RELATED HOMEOBOX11 (WOX11) and WOX12 [4], interact with AUXIN RESPONSE FACTOR7 (ARF7) and ARF19 [5] to activate *LATERAL ORGAN BOUNDARIES DOMAINs* (*LBDs*) [6, 7], allowing callus initiation and proliferation. Competence for regeneration in the callus is established by root stem cell regulators, including PLETHORAs (PLTs), SCARECROW (SCR), and WOX5 [3, 8, 9]. Following pluripotency acquisition, de novo shoot regeneration can be facilitated by incubating the callus on SIM. Type-B ARABIDOPSIS RESPONSE REGULATORs (ARRs), which promote cytokinin signaling, act as transcriptional activators of *WUSCHEL* (*WUS*), a core regulator for the establishment of the shoot meristem [10, 11].

Cytosine methylation is a fundamental epigenetic modification critical for silencing transposable elements (TEs), maintaining genome stability, and regulating gene expression in plants and vertebrates [12–14]. Unlike mammals, plants methylate not only at CG sites but also at CHG and CHH sites, albeit to a lesser extent than CG sites [14]. In *Arabidopsis thaliana*, de novo DNA methylation in all sequence contexts is facilitated by the DOMAIN REARRANGED METHYLTRANSFERASE1/2 (DRM1/2) through the small RNA-directed DNA methylation (RdDM) pathway [15, 16]. METHYLTRANSFERASE1 (MET1) maintains CG DNA methylation [17], whereas CHROMOMETHYLASE3 (CMT3) and CMT2 contribute to the maintenance of CHG and CHH methylation, respectively [14, 18, 19].

While DNA methylation patterns are maintained during cell division, ensuring genome stability and preserving cell lineage, they also undergo dynamic reprogramming, which involves active demethylation during development [20–27]. In *Arabidopsis*, DNA methylation across all sequence contexts can be reversibly removed by DNA glycosylase-domain proteins, such as DEMETER (DME), DEMETER-LIKE2 (DML2), DML3, and REPRESSOR OF SILENCING1 (ROS1) via the base excision repair pathway [28–30]. Intriguingly, while the *ros1;dml2;dml3* triple mutants developed fairly normally, *dme* mutants display pleiotropic developmental phenotypes, such as seed abortion and stunted growth [31], indicating that the DME demethylase is essential for both reproduction and sporophytic growth [32]. However, the full extent of DME demethylase activity and its epigenomic effects during vegetative growth remain unknown.

Although several studies have demonstrated DNA methylation changes during plant regeneration [33–36], the comprehensive landscape during two-step plant regeneration and the underlying molecular mechanisms governing its dynamic changes remain elusive. In this study, we demonstrate dynamic changes in both methylome and transcriptome throughout the tissue culture process in WT L*er* and *dme-2* mutants. We further reveal that the DME demethylase plays a critical role in preserving non-CG methylation in pericentromeric regions, by promoting the RdDM pathway during regeneration. Our results highlight not only the close relationship between DME-mediated DNA demethylation and RdDM, but also the global dynamics of DNA methylation during plant regeneration, providing key insights into epigenetic reconfiguration that occur for somatic cell reprogramming.

## Results

### Global DNA methylation dynamics during two-step regeneration process

**Fig. 1:**
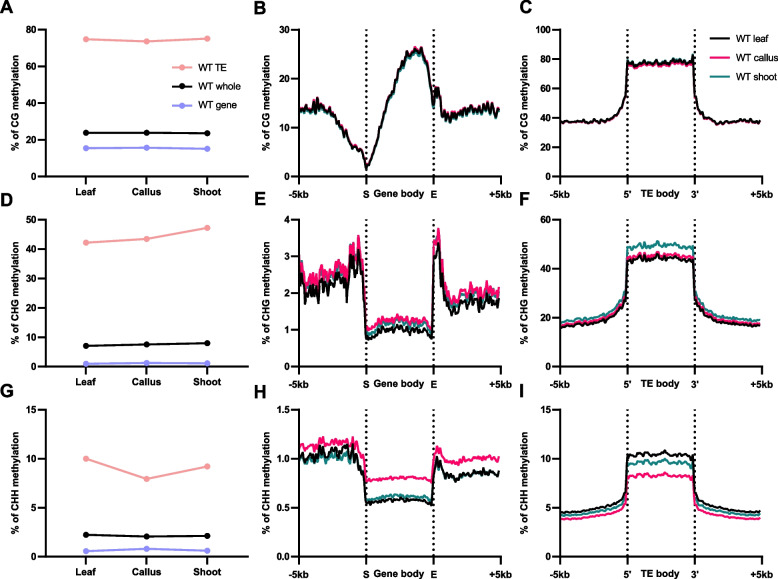
DNA methylation levels in global and genomic features during *Arabidopsis* regeneration in L*er* wild type. The average DNA methylation levels of L*er* wild type during regeneration process in CG (**A**-**C**), CHG (**D**-**F**), and CHH (**G**-**I**) contexts by different genomic features **A**, **D**, and **G**, whole genome, gene, and transposable elements in leaf, callus, and de novo shoot. DNA methylation levels of body of genes (**B**, **E**, and **H**) and TEs (**C**, **F**, and **I**), as well as their surrounding regions are shown

Two-step plant regeneration is widely used for studying somatic cell reprogramming. Using *Arabidopsis* L*er* leaf as explants, calli were induced on CIM for 7 days. Then, calli were transferred onto SIM to facilitate the regeneration of de novo shoots (Additional file 1: Fig. S1). To investigate the DNA methylation dynamics during the regeneration process, whole genome bisulfite sequencing (WGBS) was performed on leaf explants, DAC7 (7 days after incubation on CIM) calli, and DAS14 (14 days after incubation on SIM) de novo regenerated shoots (Additional file 1: Fig. S1). The average DNA methylation levels of wild type (WT) were calculated separately for whole genome, gene and TE. In general, the average CG methylation levels remained relatively constant during the regeneration process in both genes and TEs (Fig. 1A-C). In contrast, a gradual increase in CHG methylation levels was observed, especially in TEs, during both callus formation and shoot regeneration (Fig. 1D-F).

Notably, CHH methylation levels in TEs exhibited a dynamic pattern dependent on the tissue culture step: it gradually decreased during callus formation on CIM, but increased in de novo regenerated shoots induced from callus on SIM (Fig. 1G-I). The observed dynamics of CHH methylation in TEs during two-step plant regeneration resembles what occurs during embryogenesis, as CHH methylation levels are low in early embryos but increase as embryos undergo organogenesis and mature [37–41]. The shared CHH methylation dynamics in calli and early embryos suggest a potential role of global DNA methylation reprogramming, particularly at CHH sites, in cellular pluripotency.

### Substantial changes in DNA methylation landscapes during plant regeneration process

**Fig. 2:**
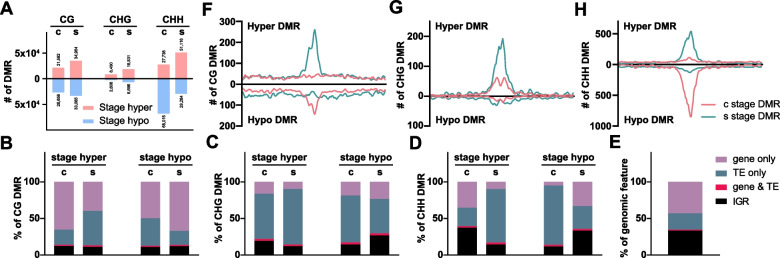
The number, composition, and positions on chromosome 1 of each stage’s DMRs during plant regeneration. **A** The total number of stage DMRs. **B**-**D** The composition of stage DMRs (gene, gene & TE, TE, and IGR). **E** The composition of a reference *Arabidopsis* Ler genome for a control. **F**-**H**, The positions of stage DMRs were analyzed on chromosome 1. The size of a window is 50 bp. c stage DMR, DMR extracted from leaf-to-callus transition (7 days on CIM); s stage DMR, DMR extracted from callus-to shoots transition (14 days on SIM); IGR, intergenic regions; gene & TE, the window probe overlapped with both the gene and the TE

To investigate the extent of methylation differences during regeneration and characterize these sites, stage-specific differentially methylated regions (DMRs) were identified. DMRs during leaf-to-callus transition were designated as 'c stage DMRs', while DMRs during callus-to-shoot formation were designated as 's stage DMRs'. During the leaf-to-callus transition, while global CG methylation levels remained relatively stable (Fig. 1A), a substantial number of both CG hyper DMRs and hypo DMRs were identified (21,592 hyper and 26,659 hypo, respectively) (‘c’ in Fig. 2A). Notably, CG hyper DMRs were predominantly enriched in genic regions, whereas CG hypo DMRs were found in roughly equal proportions within both gene and TE regions (Fig. 2B and E). Interestingly, CG hyper DMRs are evenly distributed throughout chromosomes, whereas CG hypo DMRs show a peak in pericentromeric regions (Fig. 2F, pink), suggesting the genome-wide redistribution of CG methylation in genic regions, along with substantial CG hypomethylation in pericentromeric TEs, during leaf-to-callus formation.

When the callus was transferred to SIM, the number of CG DMRs significantly increased (Fig. 2A). While the majority of the de novo shoot CG hyper DMRs were enriched in pericentromeric TEs, CG hypo DMRs were located in genic regions (Fig. 2B, E, and F, green), suggesting that during de novo shoot formation, there is a notable CG resetting in pericentromeric TEs that were hypomethylated during callus formation, accompanied by CG hypomethylation within genes (Additional file 1: Fig. S2).

Non-CG methylation was heavily enriched at TEs (Fig. 2C-E) and showed a striking enrichment at the pericentromeric region for both hyper and hypo DMRs (Fig. 2G and H). For CHG methylation, pericentromeric hyper DMRs were dominant, especially during shoot formation (Fig. 2A, C, and G). Meanwhile, during leaf-to-callus transition, the number of CHH hypo DMRs (68,015) was significantly greater than CHH hyper DMRs (27,738), resulting in global CHH hypomethylation (Figs. 1G and 2A, CHH), mostly at pericentromeric TEs (Fig. 2D and H), suggesting that callus induction from differentiated leaf tissues promotes epigenetic CHH reprogramming. However, when callus was transferred to SIM, the dynamics of CHH methylation DMRs were reversed (Additional file 1: Fig. S3), consistent with the global increase in CHH methylation (Fig. 1G-I). The s stage DMRs showed a significantly large number of CHH hyper DMRs (51,116 in Fig. 2A, CHH), which were enriched in pericentromeric TEs (Fig. 2D and H). These results indicate massive genome-wide DNA methylation landscape changes during two-step plant tissue regeneration, particularly with global CHH methylation reprogramming occurring during dedifferentiation, followed by subsequent recovery during shoot differentiation from callus.

### Global hypomethylation in pericentromeric regions during shoot regeneration in *dme-2*

**Fig. 3:**
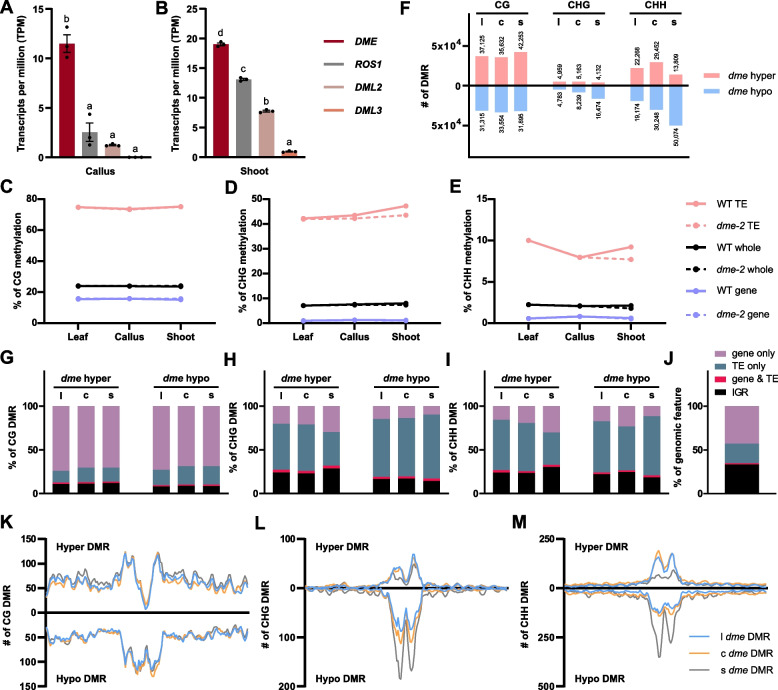
*dme-2* mutant exhibits hypomethylation in non-CG contexts, particularly enriched in TEs. **A**, **B** Expression of DNA demethylase genes in RNA-seq of WT callus and de novo shoot. Gene expression values are shown as TPM values from the RNA-seq data. Data represent mean ± SEM of three biological replicates. Significant differences were determined using one-way ANOVA, followed by Tukey’s post hoc test. Different letters indicate significant differences (*p*-value < 0.05). **C**-**E** The average DNA methylation levels of L*er* wild type and *dme-2* mutant during regeneration process in CG, CHG, and CHH contexts by different genomic features. **F** The number of *dme-2* DMRs at each stage of regeneration. **G**-**I** The composition of *dme-2* DMRs (gene, gene & TE, TE, and IGR). **J** The composition of a reference *Arabidopsis* Ler genome for a control. IGR, intergenic regions; gene & TE, the window probe overlapped with both the gene and the TE; l; leaf explant, c; callus, s; de novo shoot. **K**-**M** The positions of *dme-2* DMRs (dme hyper and dme hypo at each stage) were analyzed in a chromosomal view in CG, CHG, and CHH contexts

Since global methylation changes take place during WT callus formation and shoot regeneration (Fig. 1), we hypothesize that active demethylation may play a role in DNA hypomethylation over the regeneration process. In support, *DEMETER* (*DME*) displayed the highest expression in DAC7 callus and DAS14 shoots compared to other DNA demethylase genes (Fig. 3A, B). Moreover, *dme-2* mutants produced a significantly higher number of calli and de novo shoots compared to the WT [32]. These observations suggest that DME might play an important role during plant regeneration.

Next, we investigated genome-wide DNA methylation levels in *dme*-2 mutants compared to those in WT. Surprisingly, *dme-2* mutants exhibited global hypomethylation, especially at non-CG contexts during the regeneration process (Fig. 3C-E and Additional file 1: Fig. S4). This was particularly pronounced in CHH context at TEs during shoot regeneration (Fig. 3E). We then examined DMRs between WT and *dme-2* mutants at each stage. Although the average CG methylation levels were similar (Fig. 3C), large numbers of both hyper and hypo CG DMRs were obtained at each stage, with over 70% originating from genic regions (Fig. 3F, G, and J, CG) and distributed across chromosomal arms (Fig. 3K). This suggests that DME has pivotal roles during the entire regeneration process by regulating proper CG methylation within genes. Conversely, it is notable that *dme* CG hypo DMRs were also substantially clustered in pericentromeric regions, indicating a potential indirect effect caused by *dme-2* mutation (Fig. 3K).

Compared to the gradual increase observed in CHG methylation levels during regeneration in WT, *dme-2* mutants exhibited relatively stable CHG levels, resulting in hypomethylation at CHG context (Fig. 3D). Consistent with this, the majority of *dme* CHG DMRs were hypo DMRs (Fig. 3F, CHG), enriched at pericentromeric TEs (Fig. 3H and L). For CHH context, while the WT exhibited a dynamic decrease followed by an increase in CHH methylation during regeneration process, *dme-2* mutant displayed a consistent decrease, resulting in hypomethylated CHH methylation, especially in de novo shoots (Fig. 3E). In particular, the majority of *dme* CHH DMRs were hypo DMRs in de novo shoots (50,074 in Fig. 3F, CHH). These DMRs primarily originated from TEs (Fig. 3I), concentrated in pericentromeric regions (Fig. 3M).

It has been known that DME-mediated demethylation primarily targets short euchromatic AT-rich TEs as well as the boundaries of long TEs [21, 42]. In line with this, we observed a tendency for short (< 500 bp) TEs being more enriched in *dme* hyper CG DMRs, while long (> 2000 bp) TEs exhibited enrichment in *dme* hypo DMRs across all cytosine context (Additional file 1: Fig. S5). Consistently, CG hyper DMRs were observed in the chromosomal arms, while hypo *dme* DMRs were predominantly concentrated in pericentromeric regions (Fig. 3K) where long TEs are enriched. Altogether, our results suggest that DME is required not only for demethylating CG levels at genic regions, but also maintaining methylation levels, primarily for non-CG and, to a less extent, for CG, in pericentromeric TEs throughout the entire regeneration process.

### The role of DME in maintaining pericentromeric methylation possibly through RdDM pathway

**Fig. 4:**
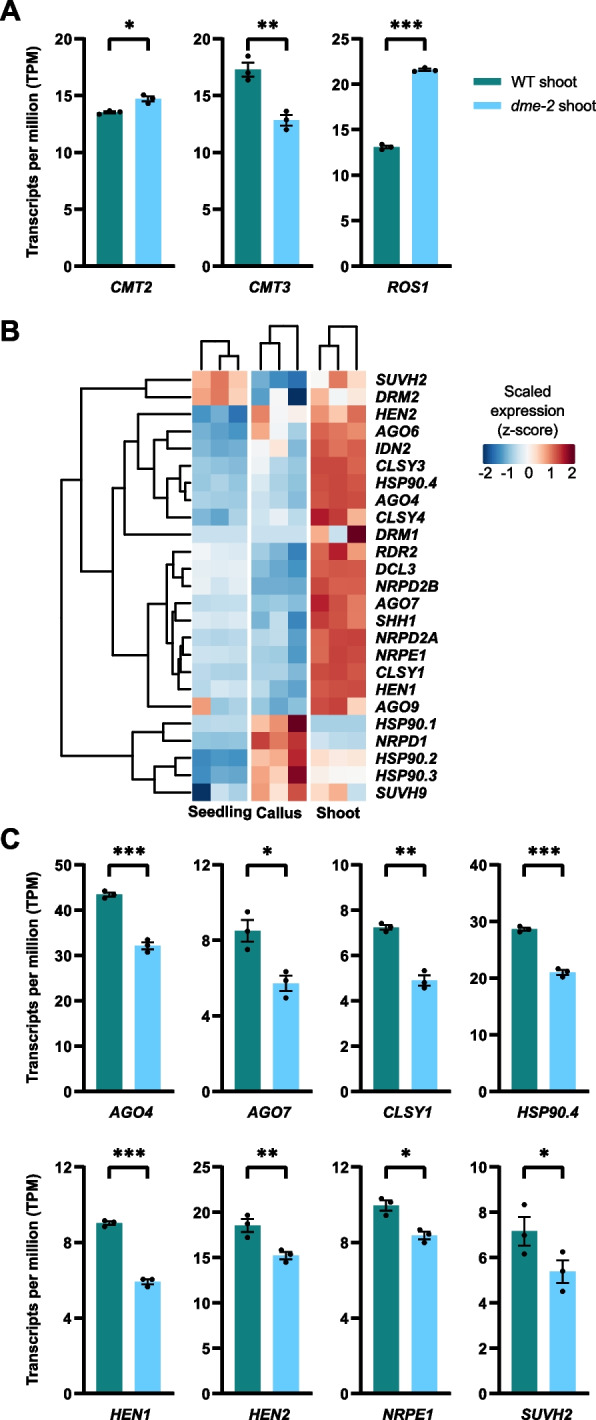
Activation of RdDM pathway during de novo shoot regeneration is inhibited in *dme-2* mutant. **A** Expression of DNA methyltransferase, *CMT2*, *CMT3*, and DNA demethylase *ROS1* in de novo shoots of WT and *dme-2*. Gene expression values are shown as TPM values from the RNA-seq data. **B** Heatmap representation of the expression pattern of genes involved in the RdDM pathway in WT seedlings, calli, and de novo shoots. Gene expression is shown as scaled TPM values from the RNA-seq data. **C** Expression of genes involved in the RdDM pathway in de novo shoots of WT and *dme-2*. (**A** and **C**) Data represent mean ± SEM of three biological replicates. Statistically significant differences were determined with DEseq2 (*p-value < 0.05, **p-value < 0.01, ***p-value < 0.001)

Given that DME functions as a DNA demethylase, it is intriguing to note the global hypomethylation in pericentromeric regions of *dme-2* mutants, which could be an indirect effect of *dme* mutation. Since pericentromeric non-CG methylation is regulated either by RNA-directed DNA methylation (RdDM) or by RNA-independent CMT3 (CHG) and CMT2 (CHH) activity, we first examined *CMT3* and *CMT2* expression in *dme-2* shoots and found that *CMT3* was decreased but *CMT2* was marginally increased in *dme-2* mutant (Fig. 4A). This rules out the possibility that CHH hypomethylation in *dme-2* mutants derives from decreased CMT2 activity. In addition, *ROS1* was highly increased by *dme* mutation in de novo shoots (Fig. 4A), which can explain the hypomethylation in *dme-2* (Fig. 3F). However, many of the key genes involved in RdDM pathway, which were highly induced during WT de novo shoot regeneration (Fig. 4B), were down-regulated in *dme-2 *de novo shoots (Fig. 4C and Additional file 1: Fig. S6), consistent with the fact that the resetting of non-CG methylation in pericentromeric TE regions during WT shoot formation is attributed at least in part by the RdDM pathway [43]. Considering that CHH reconfiguration is a characteristic feature of the reprogrammed state, the hypo CHH methylation levels observed in pericentromeric regions of the *dme-2* shoots may imply its higher cellular competence for cell fate transition, reflecting the high regeneration potential of *dme-2* even in regenerated, differentiated tissues (Additional file 1: Fig. S7). Overall, our results suggest that the DME-activated RdDM may play a critical role in the global reestablishment of DNA methylation, particularly at non-CG methylation, during shoot regeneration.

### Enhanced regeneration in *dme-2* mutant is associated with the misregulation of key genes

**Fig. 5:**
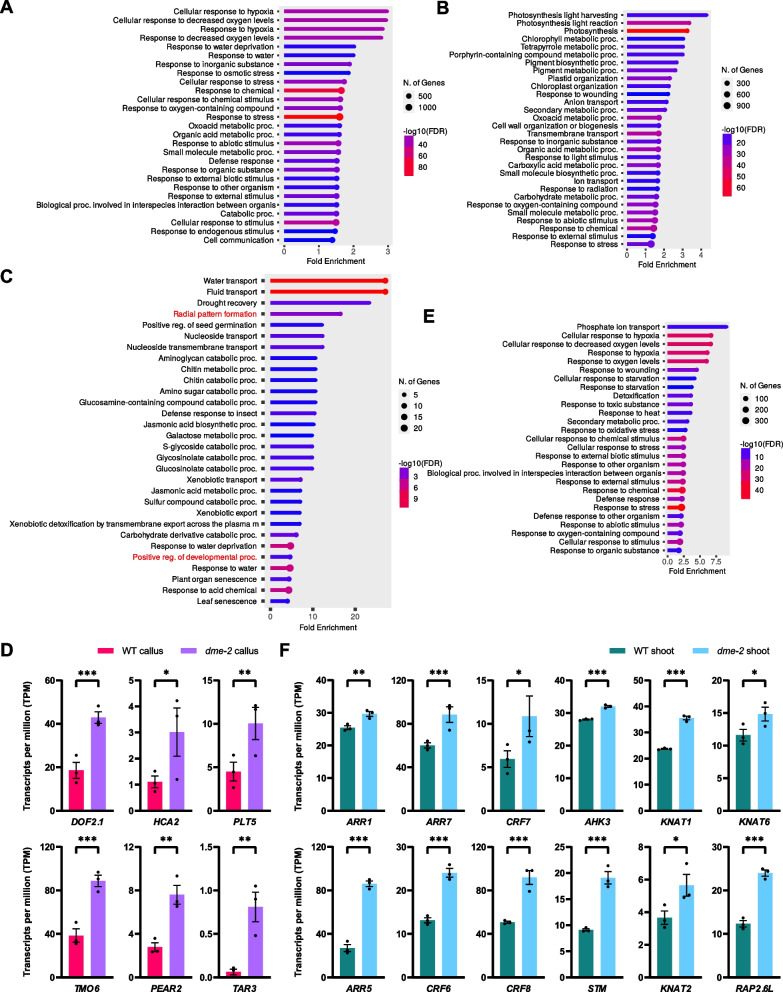
Expression of genes regulating callus formation and shoot regeneration are promoted in *dme-2* mutant. **A** Gene ontology (GO) terms enriched in genes up-regulated in calli relative to seedlings. **B** GO terms enriched in genes up-regulated in de novo shoots relative to calli. **C** GO terms enriched in genes up-regulated in *dme-2* callus relative to WT calli. **D** Expression of genes related to wound-induced tissue repair, callus formation, and pluripotency acquisition in RNA-seq of WT and *dme-2* calli. **E** GO terms enriched in genes up-regulated in *dme-2 *de novo shoots relative to WT de novo shoots. **F** Expression of genes related to cytokinin signaling, biosynthesis, and shoot stem cell maintenance in RNA-seq of WT and *dme-2 *de novo shoots. **D** and **F** Data represent mean ± SEM of three biological replicates. Statistically significant differences were determined with DEseq2 (*p-value < 0.05, **p-value < 0.01, ***p-value < 0.001)

We previously uncovered that *dme-2* mutants exhibit accelerated callus formation and enhanced shoot regeneration [32], possibly due to local DNA methylation changes affecting critical DME target genes, as well as the global redistribution of DNA methylation. To understand the molecular details underlying the DME-regulated plant regeneration, we analyzed transcriptome data for callus formation and subsequent shoot regeneration in WT and *dme-2* mutant. In WT, differential expression analysis between each stage revealed dynamic gene expression, with 11,220 differentially expressed genes (DEGs) between original seedlings and callus, and 10,748 DEGs between callus and regenerated shoots (Additional file 2: Supplementary Dataset S1). Gene Ontology (GO) enrichment analysis showed that during callus reprogramming process, GO terms such as “callus formation” and “lateral root development” were enriched particularly in genes up-regulated during callus formation (Fig. 5A and Additional file 2: Supplementary Dataset S1). Meanwhile, during de novo shoot regeneration from callus, genes related to photosynthesis were activated (Fig. 5B), along with GO terms including “shoot system morphogenesis” and “cytokinin biosynthetic process” (Additional file 2: Supplementary Dataset S1).

However, the *dme-2* mutant exhibited significant changes throughout the regeneration process. 518 DEGs were identified between WT and *dme-2* calli (Additional file 3: Supplementary Dataset S2). Among the 260 genes up-regulated in *dme-2* calli, we found the enriched GO terms "root radial pattern formation" and "positive regulation of developmental process", which could potentially be involved in the processes of cellular reprogramming and the formation of root-stem-like callus in *dme-2* mutants (Fig. 5C). DNA BINDING WITH ONE FINGER (DOF) transcription factor genes such as *DOF2.1*, *HIGH CAMBIAL ACTIVITY2* (*HCA2*), *TARGET OF MONOPTEROS6* (*TMO6*) and *PHLOEM EARLY DOF2* (*PEAR2*), which are known to play crucial roles in tissue repair in response to wounding and callus formation [44], were up-regulated in *dme-2* (Fig. 5D). Furthermore, genes essential for pluripotency acquisition, *TRYPTOPHAN AMINOTRANSFERASE RELATED3* (*TAR3*) and *PLT5*, were also up-regulated in *dme-2* calli (Fig. 5D), accounting for enhanced callus proliferation and pluripotency acquisition.

The number of DEGs between WT and *dme-2* increased in de novo shoots (1,459 DEGs) compared to that in calli (Additional file 3: Supplementary Dataset S2). GO terms related to hypoxia were enriched in genes up-regulated in *dme-2 *de novo shoots (Fig. 5E), which suggests increased cell proliferation, consistent with the enhanced cell division in endosperm [31] or stomata precursor cells [32] observed in *dme-2* mutants. Cytokinin signaling components, including *ARABIDOPSIS HISTIDINE KINASE3* (*AHK3*), *ARR1*, *ARR5*, and *ARR7*, *CYTOKININ RESPONSE FACTOR6* (*CRF6*), *CRF7*, and *CRF8* [45], were up-regulated in *dme-2* shoots (Fig. 5F). Furthermore, Class I KNOTTED1-like homeobox (KNOXI) genes, *SHOOT MERISTEMLESS* (*STM*), *KNOTTED-LIKE FROM ARABIDOPSIS THALIANA1* (*KNAT1*), *KNAT2* and *KNAT6*, which play a key role in stem cell maintenance and cytokinin biosynthesis [46–48], were also up-regulated in *dme-2 *de novo shoots (Fig. 5F). These observations indicate that *dme* mutation enhances callus induction, pluripotency acquisition, and the shoot regeneration processes (Additional file 1: Fig. S7), likely due to altered gene expression regulated by DME, both directly and indirectly.

## Discussion

The two-step plant regeneration process involves somatic cell reprogramming and tissue identity transition, and requires dynamic alterations in epigenetic landscape, including DNA methylation. Although studies suggest a linkage of DNA methylation and plant regeneration [49–52], it is elusive how dynamic DNA methylation is regulated throughout the entire tissue culture process. Our analyses confirm that during callus formation, pericentromeric TE regions experience hypomethylation primarily in the CHH context, with a lesser degree in the CG context, alongside CG redistribution within genic regions, which might play a crucial role in the transition to a reprogrammed state. During shoot regeneration, pericentromeric TE regions undergo massive methylation across all cytosine contexts, along with more dynamic redistribution in the genic CG methylation compared to what occurs during callus formation. These epigenetic changes might be a hallmark critical for the transition from reprogrammed to differentiated state.

Due to the technical challenges in obtaining a global methylome from the egg cell, zygote, and embryo immediately after fertilization, the dynamics of CHH methylation remain speculative. However, the observed CHH methylation changes during plant regeneration seem to share some similarities with CG methylation erasure and resetting in the mammalian life cycle, albeit to a lesser extent [53, 54]. Similarly, lower CHH levels are observed in early globular embryos, with a gradual increase in CHH methylation during *Arabidopsis* embryogenesis [37–41]. Therefore, plant regeneration and embryogenesis share not only common phenotypic development engaging pluripotency but also epigenetic reconfiguration. As the dynamic erasure and resetting of CG methylation during the life cycle is critical for successful mammalian gamete formation and embryogenesis, it has long been thought that a similar phenomenon could be observed in plants. Although the average CG methylation levels are significantly higher than non-CG methylation and appear to be important for gene and TE regulation in plants as in animals, the average CG methylation levels stay relatively constant. Instead, the CHH methylation levels exhibit a similar wave of erasure and reestablishment [37–41]. Therefore, it is plausible to suggest that the global CHH methylation changes in pericentromeric regions might be important epigenetic processes during cell fate transitions. Epigenetic reprogramming during callus formation may contribute to acquiring cellular competence for de novoorganogenesis, while the reestablishment of DNA methylation may help shape specific tissue identity.

DME plays a pivotal role in the global resetting of DNA methylation during de novo shoot regeneration. Considering that DME is a demethylase acting in all cytosine contexts [28], it was unexpected to observe global hypomethylation, especially in the pericentromeric regions in *dme-2* mutants. Our data suggest that increased ROS1 demethylase activity, as well as an inefficient RdDM pathway including DRM1/2, might be the reasons for this. Pericentromeric hypomethylation in *dme-2* mutants represents a more reprogrammed state compared to WT during regeneration process, thereby generating more callus and shoots, in addition to enhancing expression of the core regulators of pluripotency acquisition and shoot meristem development. Consistent with this, *drm1/2* mutants showed increased callus formation upon CIM induction compared to WT [55]*.* Given the interplay between ROS1 and RdDM activity in maintaining proper DNA methylation levels [56, 57], it is plausible that DME may contribute to ROS1-RdDM activity during sporophytic development. This hypothesis is supported by observations that *dme* mutants exhibit upregulation of *ROS1* expression and downregulation of key RdDM genes, although the molecular mechanism remains unknown. DNA methylation and demethylation are intimately balanced though their coordinated feedback regulation to maintain the overall DNA methylation levels in plants. It is currently elusive how DNA methylation is reprogrammed during callus formation. It might be driven by active cell proliferation as well as reduction in small RNA population. Future studies will unravel the molecular details of DNA methylation erasure and its linkage to DME-mediated DNA methylation resetting.

Taken together, it is noteworthy that DNA methylation reconfiguration is important for cellular pluripotency and differentiation in the course of plant regeneration. Global DNA hypomethylation during callus formation might lead to TE activation, which can induce unpredictable, massive changes in genome structures during in vitro tissue culture [58]. Our findings demonstrate that DME-induced RdDM activation is a crucial genetic and epigenetic program that might safeguard genome stability, minimizing genome destruction during plant tissue culture.

## Conclusion

Plant cells are capable of acquiring pluripotency and regenerating. In this study, we demonstrate that global reconfiguration of DNA methylation, accompanied by transcriptome changes, occurs during the two-step plant regeneration process. During callus formation, substantial CG and extensive CHH hypomethylation occur in pericentromeric regions, establishing cellular competence for regeneration. Upon de novo shoot regeneration from callus, pericentromeric methylation levels significantly increase across all cytosine contexts, likely driven by the RdDM pathway. The DME demethylase plays crucial roles in global redistribution of CG methylation, as well as in maintaining non-CG methylation in pericentromeric regions during shoot regeneration. DME is essential for the RdDM process, as the *dme-2* mutant exhibits significant non-CG hypomethylation with low RdDM activity, resulting in a more pluripotent state that produces more callus and regenerated shoots. In addition to pericentromeric DNA methylation, CG methylation is extensively reconfigured across genic regions throughout the regeneration process, with DME regulating key genes involved in pluripotency establishment and de novo organogenesis, thereby contributing to efficient somatic cell reprogramming. Our study reveals dynamic changes in the epigenetic landscape during somatic cell reprogramming and subsequent regeneration into new cell fates. It also highlights the dual epigenetic role of DME in cell reprogramming: reconfiguration of genic CG methylation and global resetting of pericentromeric non-CG methylation.

## Methods and materials

### Plant materials and growth conditions

The L*er* ecotype *Arabidopsis thaliana* WT and *dme-2* mutant [31] were utilized for plant regeneration in this study. Seeds were germinated on MS medium [59] and plants were grown in a controlled environment room with a long-day photoperiod (16 h light, 8 h dark) at 22 °C under cool white fluorescent light (100 $\upmu$mol/m^2^/s).

The true leaves of seedlings at 14 days after germination (DAG14) were used as explants for callus induction. Callus was induced on CIM (MS medium supplemented with 0.5 μg/ml 2,4-dichlorophenoxyacetic acid [2,4-D] and 0.05 μg/ml kinetin) at 22 °C under continuous dark conditions for 7 days. The callus at day 7 (DAC7) was then transferred to SIM (MS medium supplemented with 0.9 μmol/l 3-indoleacetic acid, 2.5 μmol/l 2-isopentenyl adenine) and cultured for up to 2 weeks at 22 °C under long-day conditions to induce de novo shoot.

### Sample preparation for WGBS and transcriptome

All samples were rapidly frozen using liquid nitrogen and stored at -80 °C. Four biological replicates of leaf explant and de novo shoot, and three replicates of callus were prepared for WGBS. For all RNA analyses, we employed three biological replicates. DNA preparation followed the protocol outlined by Allen et al. (2006) [60]. The Pico Methyl-Seq Library Prep Kit from Zymo Research (U.S.) was used for constructing the DNA methylome, and sequencing was conducted on the HiSeqXten platform from Macrogen (Korea). For RNA extraction and transcriptome construction, Total RNA Qiagen RNeasy Kit (Qiagen, Germany) and the TruSeq stranded mRNA Kit (Illumina, U.S.) were used. Sequencing for the transcriptome was carried out using the NovaSeq 6000 platform from Macrogen (Korea).

### WGBS data processing

All sequencing procedures for WGBS were performed using the HiSeqXten platform (Macrogen, Korea). Paired-end reads of 150 bp were generated from WGBS, and all reads were trimmed (10 bp from the 5’ end and 5 bp from the 3’ end) using Trim Galore. Low-quality and short reads (< 40 bp) were then removed using Trimmomatic.

Reads were aligned to the *Arabidopsis* L*er* genome [61] (accession number GCA_900660825) using hisat2 with Bismark under the option -hisat2 -local. PCR duplicates were removed, and methylation levels were extracted using the Bismark toolset. We used 50 bp windowed average satisfying at least three cytosines with a minimum of five aligned reads in each sample. Methylation levels were calculated by dividing the counts of methylated cytosines by the total counts of cytosines at each cytosine site, and then averaged within each window. To assess the CT conversion ratio, we utilized the plastid sequence from the TAIR10 genome since there is no plastid sequence available for the L*er* genome constructed by Goel et al. (2019) [61]. Information about the methylome libraries is in Additional file 4: Table S1.

### Identification of differentially methylated region

The R package called methylKit [62] was utilized for the identification of stage DMR and dme DMR. We calculated the average and standard deviation of the difference in methylation levels for each 50 bp window. If the difference of methylation level exceeded one standard deviation within each window and the q-value was below 0.05, we classified the window as a DMR (Additional file 5: Supplementary Dataset S3).

### RNA-seq analysis

Reads were mapped to the *Arabidopsis* L*er* genome [61] (accession number GCA_900660825) using STAR (v2.7.10a) with “–pOverlapNbasesMin 12 –peOverlapMMp 0.1 –twopassMode Basic” options [63], and RSEM (v1.3.1) was used to quantify transcript abundance [64]. DESeq2 (v1.34.0) was used to identify DEGs with a cut-off of log2(fold-change) > 1 or < -1 and *p*-value < 0.05 (Additional file 2: Supplementary Dataset S1 and Additional file 3: Supplementary Dataset S2) [65]. ShinyGO (v0.80) was used to perform GO enrichment analysis of the DEGs [66]. In order to perform RNA-seq analysis of L*er* and *dme-2* seedlings, raw data generated by Kim et al. (2021) [32] were downloaded from the Gene Expression Omnibus under GSE164217.

### RT-qPCR

cDNA synthesis was carried out using the QuantiTect Reverse Transcription kit (Qiagen, Germany) following the manufacturer’s instructions. Real-time qPCR was performed with the SsoAdvanced Universal SYBR Green Supermix (Bio-Rad, U.S.) and the indicated primers listed in Additional file 1: Table S2.

## Supplementary Information

Additional file 1. Figure S1 – S7, Table S2.

Additional file 2. Supplementary Dataset S1.

Additional file 3. Supplementary Dataset S2.

Additional file 4. Table S1.

Additional file 5. Supplementary Dataset S3.

## Data Availability

The data supporting the findings of this study are available within the article and its additional files. All sequencing data, raw and processed files, have been submitted to the Sequence Read Archive (http://www.ncbi.nlm.nih.gov/sra) and Gene Expression Omnibus (http://www.ncbi.nlm.nih.gov/geo) at NCBI and are publicly available as of the date of publication under PRJNA1122321 and GSE269609.
