# Supplementary material for: DEMETER DNA demethylase reshapes the global DNA methylation landscape and controls cell identity transition during plant regeneration
Source: BMC Genomics. 2024 Dec 23;25:1234. doi: 10.1186/s12864-024-11144-x (PMC11665089; doi:10.1186/s12864-024-11144-x)
Supplement: Supplementary file 1 — Additional file 1. Figure S1 – S7, Table S2. [file 12864_2024_11144_MOESM1_ESM.docx]

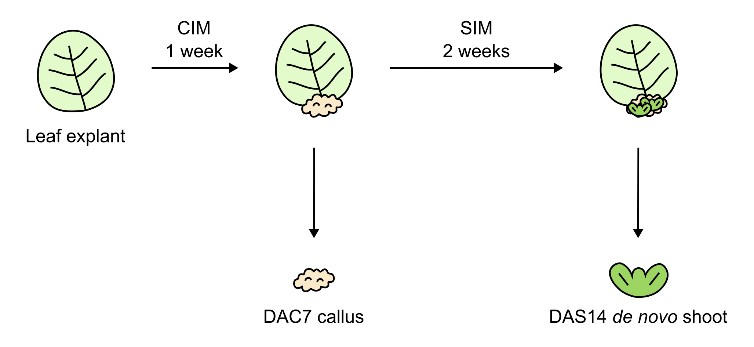


**Fig. S1** Schematic diagram of the two-step regeneration process used in this study.

*Arabidopsis* leaf explants (14 days after germination) of L*er* WT and *dme-2* mutant were used to induce callus on callus-inducing media (CIM) for 7 days. Subsequently, the calli were transferred to shoot-inducing media (SIM) to promote the regeneration of *de novo* shoots. 14 DAG Leaf explants, DAC7 callus, and DAS14 *de novo* shoots were used in this study.


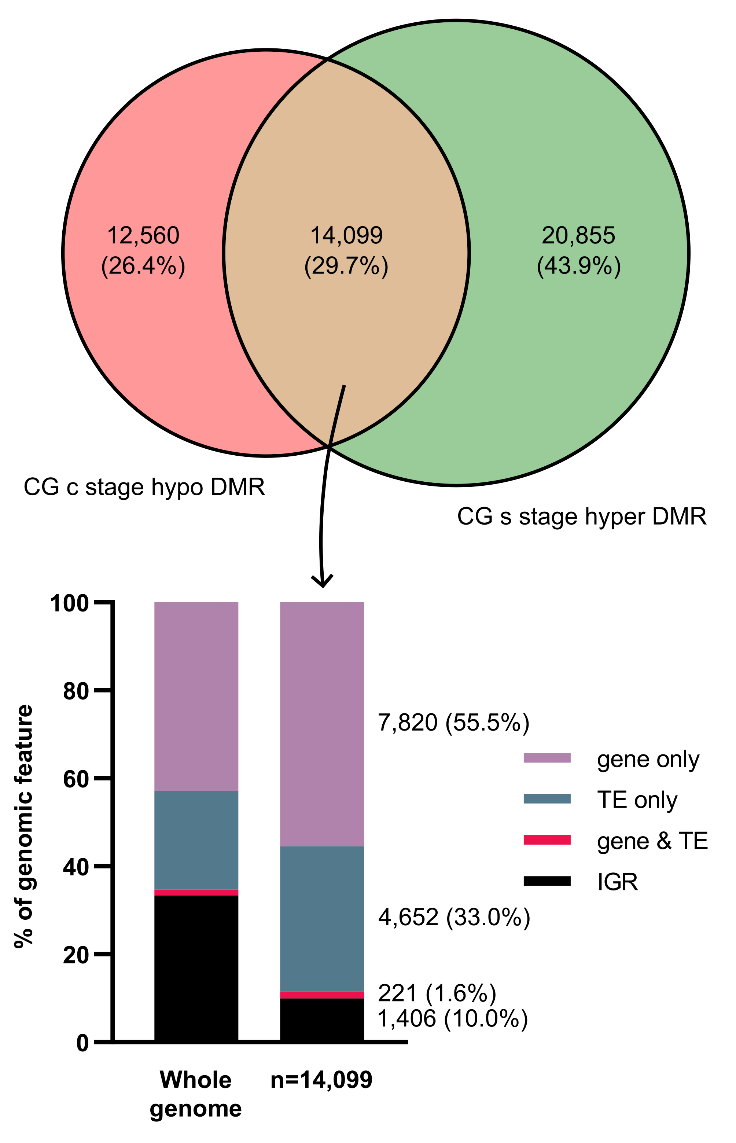


**Fig. S2** Composition of overlapping CG stage DMRs during two-step plant regeneration.

The intersection between CG methylation patterns is represented by a Venn diagram, where CG hypomethylated DMRs during leaf-to-callus transition (CG c stage hypo DMRs; pink circle, 26,659 regions) are compared with CG hypermethylated DMRs during callus-to-shoot development (CG s stage hyper DMRs; green circle, 34,954 regions). A substantial proportion (14,099 regions; 29.7%) of DMRs is shared between these DMRs. The bar graph below depicts the genomic feature composition of these overlapping regions compared to the whole genome distribution. Within the 14,099 overlapping DMRs, genic regions were predominant (7,820; 55.5%), followed by transposable elements (TEs; 4,652; 33.0%), intergenic regions (IGR; 1,406; 10.0%), and gene and TE overlapping regions (221; 1.6%).


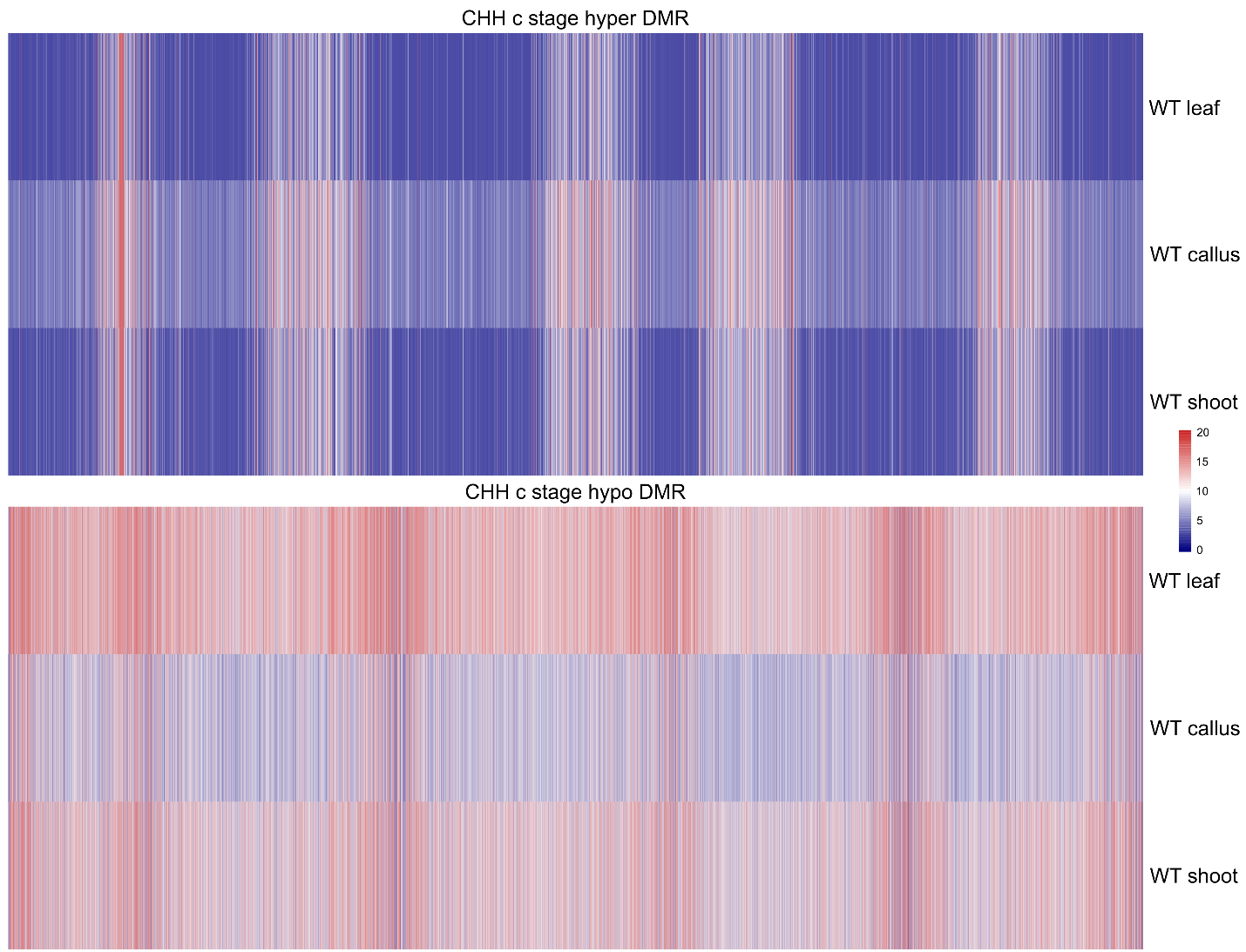


**Fig. S3** Heatmap of CHH c stage DMRs across different stages during plant regeneration.

The methylation patterns in leaf-to-callus CHH DMRs (CHH c stage DMRs) were analyzed across three developmental stages: WT leaf explants, callus, and *de novo* shoots. Color scale represents methylation levels from 0 (blue) to 20 (red). Notably, the methylation signatures in leaf explants display stronger similarity to regenerated shoots compared to intermediate callus tissue, suggesting the dynamic reversals in CHH methylation during the regeneration process. Upper panel shows hyper DMRs while lower panel represents hypo DMRs.


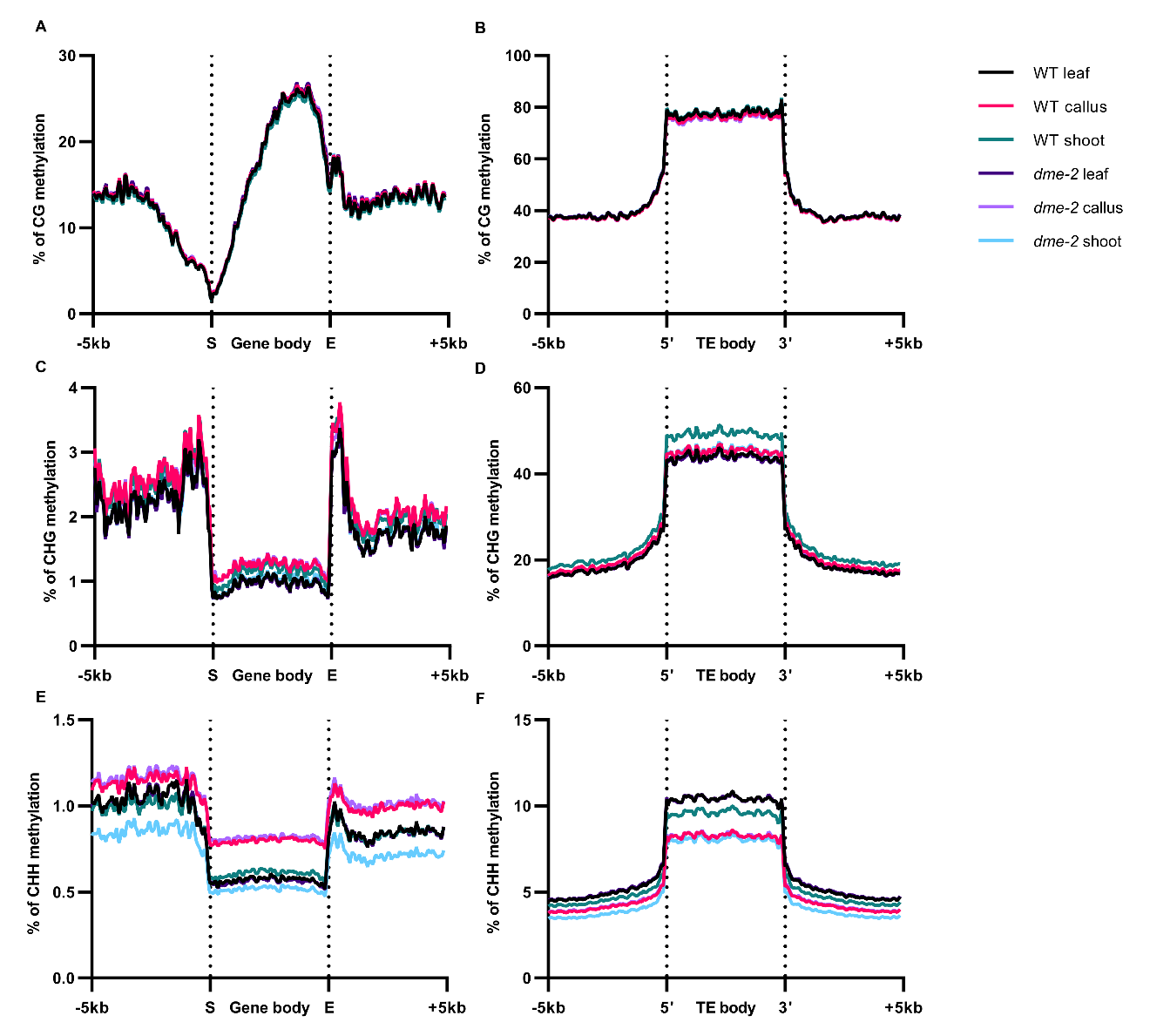


**Fig. S4** The average DNA methylation levels of genes, TEs, and their surrounding regions at each stage of regeneration in WT and *dme-2* mutants.

The average DNA methylation levels of genes, TEs, and their 5’ and 3’ surrounding regions were analyzed for each cytosine context in leaves, calli, and *de novo* shoots of WT and *dme-2* mutants.


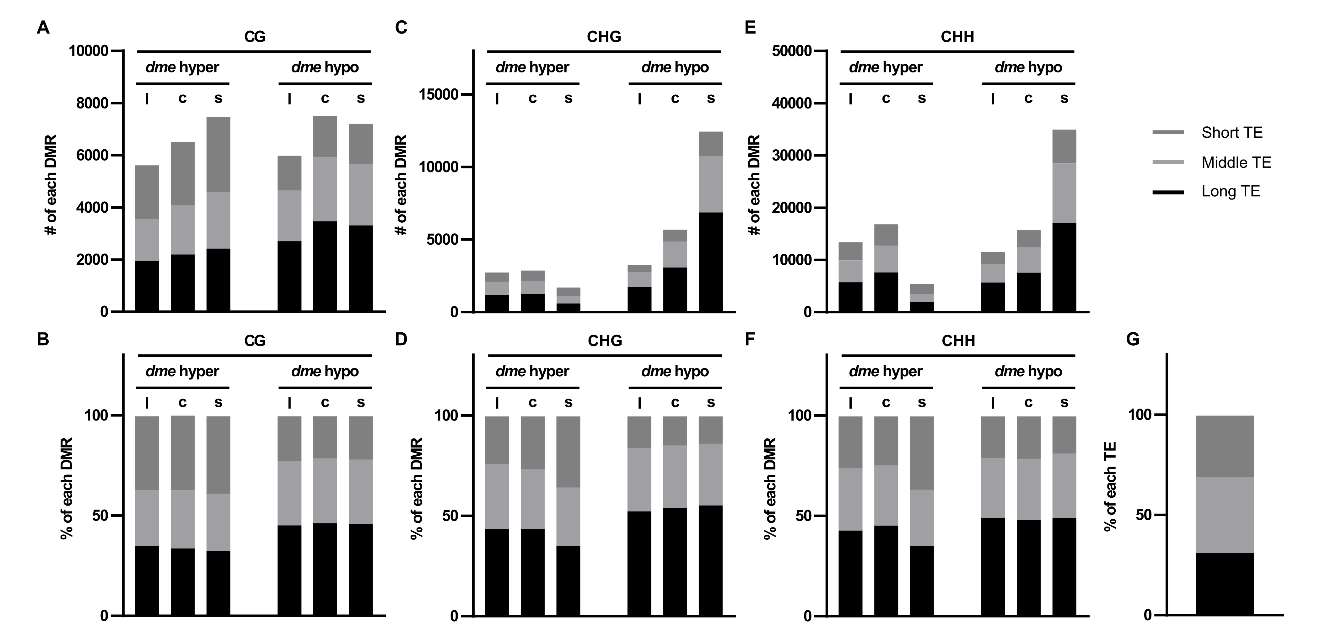


**Fig. S5** The composition of each *dme-2* DMR based on TE length.

TEs shorter than 500bp are classified as short TEs, while TEs longer than 2000bp are classified as long TEs. TEs with lengths between 500bp and 2000bp are classified as middle TEs. The number of each *dme-2* DMR based on TE length is counted (**A**, **C**, **E**), and their ratios are also calculated (**B**, **D**, **F**) in CG, CHG, and CHH contexts. **G**, The proportions of short, middle, and long TEs were calculated among all TEs in the *Arabidopsis* genome for a reference.


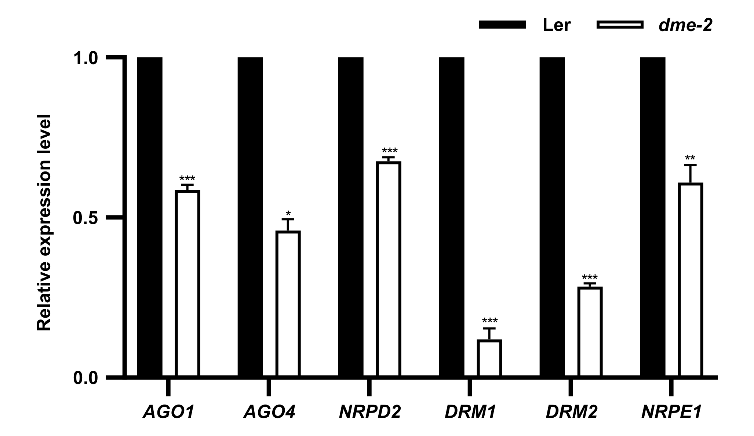


**Fig. S6** Expression levels of a few key RdDM genes in *de novo* shoots.

The relative expression levels of RdDM related genes in *de novo* shoot (DAS14) detected by real time qPCR. The vertical axis shows the relative gene expression levels as the means SE (n = 3) relative to wild-type. Statistical significance was assessed using Student’s t-test (**p*-value <0.05, ***p*-value <0.01, ****p*-value <0.001).


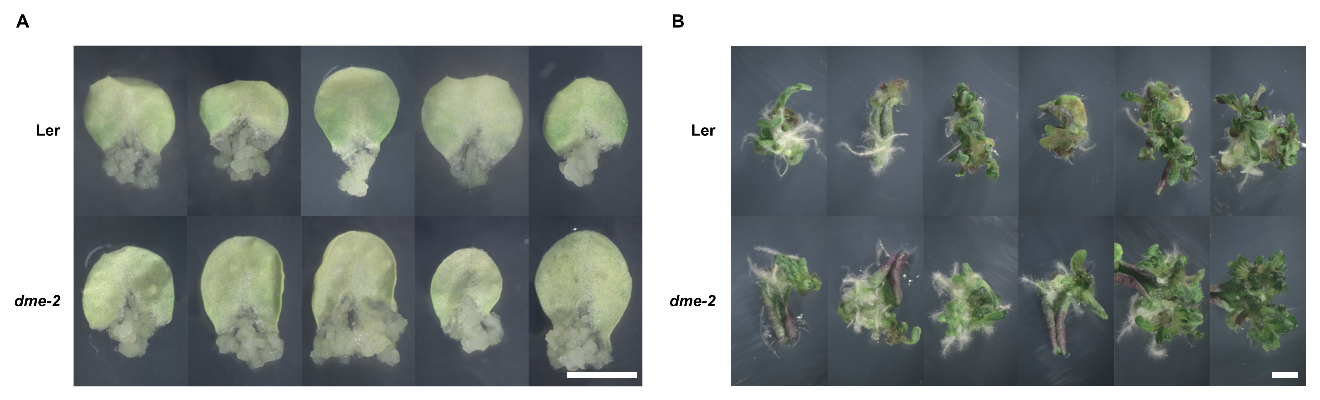


**Fig. S7** Callus and *de novo* shoot formation are enhanced in *dme-2* mutant.

**A**, Leaf explants incubated 14 days in CIM produced more calli in *dme-2* mutants. **B**, More *de novo* shoots were regenerated in *dme-2* mutant. Calli were transferred to SIM and incubated for 14 days and compared between WT and *dme-2* mutants.

**Table S2. Primers used in real-time qPCR.**

| **Primer name** | **Sequence** |
| --- | --- |
| ACT2-F | 5’-CCTTTAACTCTCCCGCTATGT-3’ |
| ACT2-R | 5’-GTAAGGTCACGTCCAGCAAG-3’ |
| AGO1-F | 5’-TCAAGCCCATCTATTGCTGC-3’ |
| AGO1-R | 5’-ATCATGCCACCAGTCACCAC-3’ |
| AGO4-F | 5’-TGGATGGTAAAGAGTTTGCT-3’ |
| AGO4-R | 5’-CCATCACTTGGACTTTCATT-3’ |
| NRPD2-F | 5’-GATGCTAGATATCCGCACCCC-3’ |
| NRPD2-R | 5’-CAGCTCTTCCATTCCACAAGC-3’ |
| DRM1-F | 5’-TAGAGCAATTGAAGAAACCGC-3’ |
| DRM1-R | 5’-CATTCGTGATCTCTCCCACATCT-3’ |
| DRM2-F | 5’-AAAATGTGGATATTGCAGAG-3’ |
| DRM2-R | 5’-TCCTATCATTGGATTTGGTA-3’ |
| NRPE1-F | 5’-CATCCGTCTGCGTACCCTG-3’ |
| NRPE1-R | 5’-TCAACCGTGATGAAGTCAACG-3’ |

Primers listed in the table were used to perform real-time qPCR assay for key RdDM genes.
